# Ultrasound-guided hydrodissection of the subclavius muscle region in neurogenic thoracic outlet syndrome: a case report

**DOI:** 10.3389/fmed.2026.1804081

**Published:** 2026-05-29

**Authors:** Yonghyun Yoon, King Hei Stanley Lam, Jason Lee, Jaewoo Lim, Jihyo Hwang, Rina Shin, Teinny Suryadi, Anwar Suhaimi, Jaeik Choi, Gyungseog Ko

**Affiliations:** 1 Department of Orthopedic Surgery, Hallym University Kangnam Sacred Heart Hospital, Yeongdeungpo, Republic of Korea; 2 International Academy of Regenerative Medicine, Incheon, Republic of Korea; 3 Incheon Terminal Orthopedic Surgery Clinic, Incheon, Republic of Korea; 4 International Association of Musculoskeletal Medicine, Kowloon, Hong Kong SAR, China; 5 MSKUS, Vista, CA, United States; 6 The Faculty of Medicine, The University of Hong Kong, Hong Kong, Hong Kong SAR, China; 7 The Faculty of Medicine, Chinese University of Hong Kong, New Territories, Hong Kong SAR, China; 8 The Hong Kong Institute of Musculoskeletal Medicine, Kowloon, Hong Kong SAR, China; 9 Hermina Podomoro Hospital, North Jakarta, Indonesia; 10 Medistra Hospital, South Jakarta, Indonesia; 11 Synergy Clinic, West Jakarta, Indonesia; 12 Department of Rehabilitation Medicine, Faculty of Medicine, Universiti Malaya, Kuala Lumpur, Malaysia; 13 Dr. Choi's Rehabilitation Clinic, Ansan, Republic of Korea; 14 Ko gyung Seog Orthopaedic Clinic, Seoul, Republic of Korea

**Keywords:** hydrodissection, muscle atrophy, neurogenic thoracic outlet syndrome, subclavius muscle, thoracic outlet syndrome, ultrasound-guided intervention

## Abstract

**Background:**

Thoracic outlet syndrome (TOS) is frequently underdiagnosed due to nonspecific symptoms and the absence of definitive radiographic findings. Neurogenic TOS (nTOS), the most common subtype, is typically caused by compression of the brachial plexus within anatomically constrained spaces. While muscular contributors such as the scalene and pectoralis minor muscles have been widely discussed, the role of the subclavius muscle remains underrecognized.

**Case presentation:**

A 53-year-old male presented with an 18-month history of progressive right upper limb weakness and medial paresthesia refractory to prior treatments for presumed cervical spondylosis. Electrodiagnostic studies demonstrated persistent proximal motor slowing of the right ulnar nerve. Ultrasonography revealed marked atrophy of the right subclavius muscle compared with the contralateral side. Ultrasound-guided hydrodissection using 30 mL of 5% dextrose in water (D5W) was performed in the infraclavicular region with particular attention to the subclavius muscle and costoclavicular space. Immediate post-procedural assessment showed greater than 70% improvement in paresthesia, with subtle objective motor improvement on bedside examination that was not yet sufficient to change the MRC grade. Following six treatment sessions, complete resolution of symptoms and full motor strength recovery were achieved.

**Conclusion:**

In a patient with unexplained proximal neuropathy, ultrasonography identified focal subclavius muscle atrophy as a potential compressive contributor within the costoclavicular space, and ultrasound-guided hydrodissection of the infraclavicular region resulted in rapid and sustained clinical improvement. While the injectate volume (30 mL) likely affected adjacent structures such as the pectoralis minor and surrounding fascial planes, this minimally invasive approach may be a feasible option for selected patients with refractory nTOS. To our knowledge, this is the first reported case of ultrasound-guided hydrodissection of the infraclavicular region with attention to the subclavius muscle for neurogenic thoracic outlet syndrome associated with muscle atrophy. Further studies are warranted to confirm efficacy and to refine patient selection criteria.

## Introduction

Thoracic outlet syndrome (TOS) comprises a heterogeneous group of disorders caused by compression of neurovascular structures as they traverse the thoracic outlet. Despite its clinical significance, TOS remains frequently underdiagnosed due to nonspecific symptoms, overlap with cervical radiculopathy and peripheral neuropathies, and the frequent absence of definitive findings on conventional imaging studies (1, 2).

TOS is commonly classified according to the primary structure involved—arterial, venous, or neurogenic—as well as the anatomical site of compression, including the interscalene triangle, costoclavicular space, and subcoracoid (pectoralis minor) space (3, 4).

Neurogenic TOS (nTOS) represents the most prevalent subtype and is primarily attributed to compression of the brachial plexus. Well-recognized contributors include cervical ribs, fibrous bands, and hypertrophy or spasm of the scalene and pectoralis minor muscles. In contrast, the subclavius muscle has received comparatively little attention, despite its intimate anatomical relationship with the brachial plexus and subclavian vessels within the costoclavicular space (5).

The subclavius muscle lies directly inferior to the clavicle and anterior to the neurovascular bundle, where even subtle pathological changes may alter the compliance and dynamics of the costoclavicular compartment (6). However, abnormalities of the subclavius muscle are easily overlooked. Its small size, oblique orientation, and limited conspicuity on routine magnetic resonance imaging or computed tomography contribute to its underrepresentation in both diagnostic algorithms and interventional strategies for nTOS. Moreover, static imaging modalities are inherently limited in assessing dynamic or posture-dependent compression, which is central to the pathophysiology of many TOS presentations.

High-resolution ultrasonography offers unique advantages in this context, allowing real-time, dynamic evaluation of the costoclavicular space and its muscular components. Ultrasound can readily identify asymmetry, architectural distortion, or atrophy of the subclavius muscle, as well as its spatial relationship to adjacent neurovascular structures. Despite these capabilities, ultrasonography has rarely been applied to specifically interrogate the subclavius muscle in patients with suspected nTOS, and interventional approaches targeting this muscle have scarcely been reported.

Ultrasound-guided hydrodissection is a minimally invasive technique in which fluid is injected to separate nerves or soft-tissue planes from surrounding fascia, muscle, or adjacent structures, with the goal of restoring tissue gliding and reducing mechanical irritation (7, 8). This approach has shown promise in various peripheral entrapment neuropathies and may also have potential utility in anatomically complex compression syndromes (7–10).

In this report, we present a novel case of neurogenic thoracic outlet syndrome associated with unilateral subclavius muscle atrophy, identified through targeted ultrasonographic assessment. The patient was successfully treated with ultrasound-guided hydrodissection of the infraclavicular region with attention to the subclavius muscle, resulting in rapid and sustained neurological recovery. This case highlights an underrecognized anatomical contributor to nTOS and underscores the diagnostic and therapeutic value of focused ultrasound-based evaluation and intervention in selected patients with refractory proximal neuropathy.

## Case presentation

### Patient history

A 53-year-old male electrician presented with an 18-month history of progressively worsening right upper-limb weakness accompanied by intermittent paresthesia along the medial forearm and hand, which substantially impaired his ability to work and participate in recreational activities, including fishing. He denied any acute traumatic event. His medical history was unremarkable except for well-controlled hypertension.

At an outside institution, his symptoms were attributed to suspected cervical spondylosis, and he underwent conservative management including physical therapy and oral medications, followed by interventional procedures such as medial branch blocks and facet joint injections. These treatments failed to provide meaningful symptomatic improvement.

Notably, he reported minimal neck pain and no radicular arm pain throughout the clinical course. Instead, his predominant complaints were progressive weakness and intermittent sensory symptoms in the medial forearm and hand. This pattern was atypical for cervical radiculopathy and raised suspicion for a proximal, extraforaminal compressive etiology.

### Diagnostic evaluation

#### Electrodiagnostic studies

Serial nerve conduction studies demonstrated persistent proximal motor slowing, most prominently affecting the right ulnar nerve above the elbow. No focal conduction block, abnormal distal latency, or sensory nerve involvement was identified at common distal entrapment sites, including the cubital tunnel and Guyon’s canal. This pattern argued against distal ulnar neuropathy and isolated cervical radiculopathy, and instead supported a proximal compressive process involving the brachial plexus (11). Key abnormal findings are summarized in Table 1.

**Table 1:** Key abnormal electrodiagnostic findings.

Date	Side	Nerve	Segment	Conduction Velocity (m/s)	Interpretation
Apr 2025	Right	Ulnar	Above elbow	24.7	Marked proximal motor slowing
Aug 2025	Right	Ulnar	Above elbow	28.2	Persistent proximal motor slowing

Normal reference values: Motor conduction velocity >50 m/s.

#### Imaging studies

Plain cervical radiographs demonstrated degenerative cervical changes without evidence of cervical ribs or elongated transverse processes (Figure 1).

**Figure 1:**
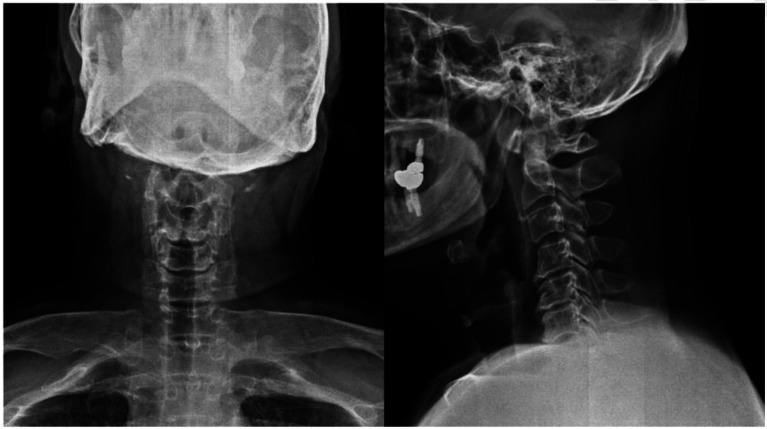
Cervical spine radiography.

Cervical spine radiograph demonstrating degenerative cervical changes without evidence of cervical ribs or elongated transverse processes.

An anteroposterior radiograph of the right elbow demonstrated degenerative bony fragments adjacent to the cubital tunnel (Figure 2A), whereas the lateral view showed preserved joint space without osseous deformity (Figure 2B). Despite these radiographic findings, distal ulnar nerve entrapment was considered clinically unlikely given the absence of sensory deficits, preserved distal nerve conduction parameters, and no symptom provocation with elbow positioning.

**Figure 2:**
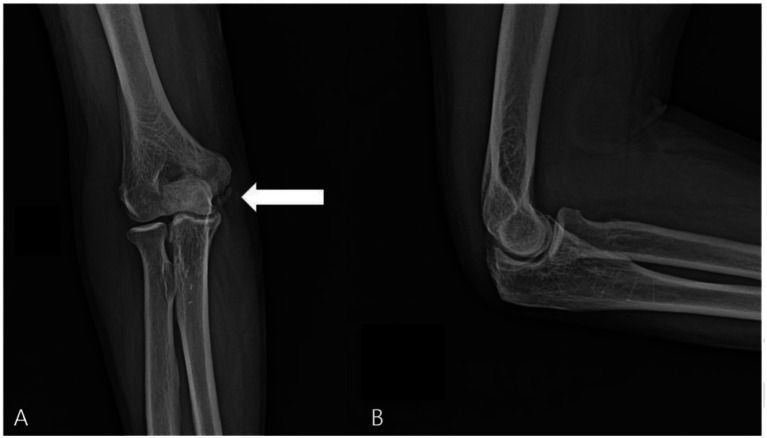
Right elbow radiography. **(A)** Anteroposterior view showing degenerative bony fragments adjacent to the cubital tunnel. **(B)** Lateral view demonstrating preserved joint space without osseous deformity.

External cervical spine magnetic resonance imaging (MRI) showed no gross spinal cord compression on T2-weighted sagittal scout images and no obvious high-grade neural foraminal stenosis on T2-weighted axial scout images (Figures 3A,B). Coronal MRI of the cervical spine and brachial plexus region demonstrated preserved continuity of the spinal cord and nerve roots, without findings suggestive of avulsion such as pseudomeningocele or gross root discontinuity (Figure 4). Collectively, these MRI findings excluded major structural causes—including cervical myelopathy, high-grade foraminal stenosis, and nerve root avulsion—and supported an extraforaminal source of neurogenic compression.

**Figure 3:**
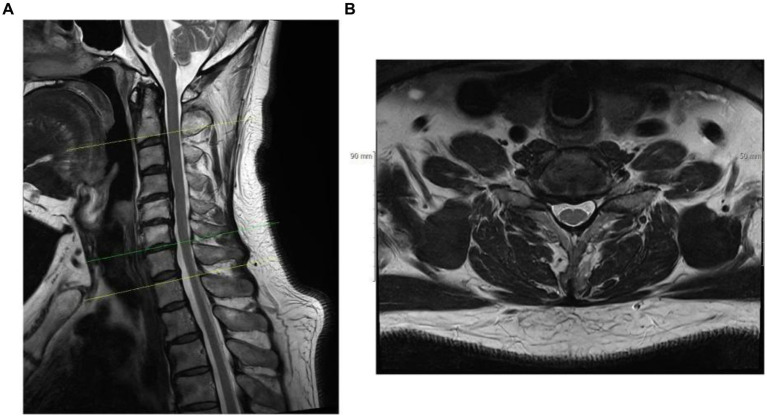
Cervical spine MRI scout images. **(A)** T2-weighted sagittal scout image showing no gross cervical spinal cord compression. **(B)** T2-weighted axial scout image showing no obvious high-grade neural foraminal stenosis.

**Figure 4:**
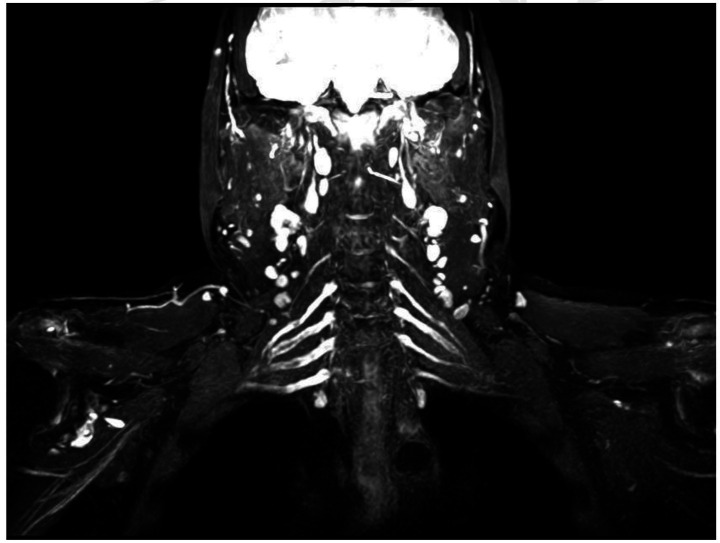
Coronal MRI of the cervical spine and brachial plexus region.

The original radiology report of the cervical and brachial plexus MRI did not comment on the subclavius muscle or the infraclavicular brachial plexus specifically, as these are not routinely assessed on standard cervical spine or brachial plexus MRI protocols. Retrospective review of the coronal and axial sequences by the authors did not reveal definitive compression of the brachial plexus by the subclavius muscle, nor were there clear structural abnormalities of the infraclavicular plexus. This highlights a limitation of conventional static MRI for evaluating dynamic or posture-dependent compression within the costoclavicular space, and underscores the complementary value of targeted ultrasonography.

Scout views show no apparent cervical spinal cord compression and no obvious high-grade foraminal narrowing.

The image demonstrates preserved continuity of the cervical spinal cord and nerve roots, with no imaging findings suggestive of spinal cord or nerve root avulsion, such as pseudomeningocele or gross root discontinuity.

### Physical examination

Physical examination revealed visible atrophy of the right elbow flexors and intrinsic hand muscles. Manual muscle testing demonstrated weakness of wrist extension, elbow flexion, and pinch grip (Medical Research Council [MRC] grade IV). Sensory examination was largely unremarkable, with no clear dermatomal or peripheral nerve sensory deficit, and significant pain was notably absent. This dissociation between prominent motor weakness and relatively preserved sensation supported a proximal neuropathic process rather than a distal entrapment neuropathy.

### Ultrasonographic assessment

The patient was examined in the supine position with a bolster placed beneath the scapular spine to optimize visualization of the costoclavicular space. High-resolution ultrasonography with Power Doppler showed a normal-appearing left subclavius muscle (Figure 5A), whereas the right subclavius muscle demonstrated marked atrophy with loss of normal architecture (Figure 5B). This pronounced asymmetry favored a chronic pathological change rather than an anatomical variant. The pectoralis major was identified superficial to the subclavius bilaterally, while the axillary artery (AA), axillary vein (AV), and brachial plexus (BP) were located deep (posterior) to the subclavius. Medially, the subclavius attached to the first rib. Given the subclavius muscle’s stabilizing role within the costoclavicular compartment, we hypothesized that these structural changes could reduce local compliance and increase the dynamic susceptibility of the adjacent brachial plexus to potential compression.

**Figure 5:**
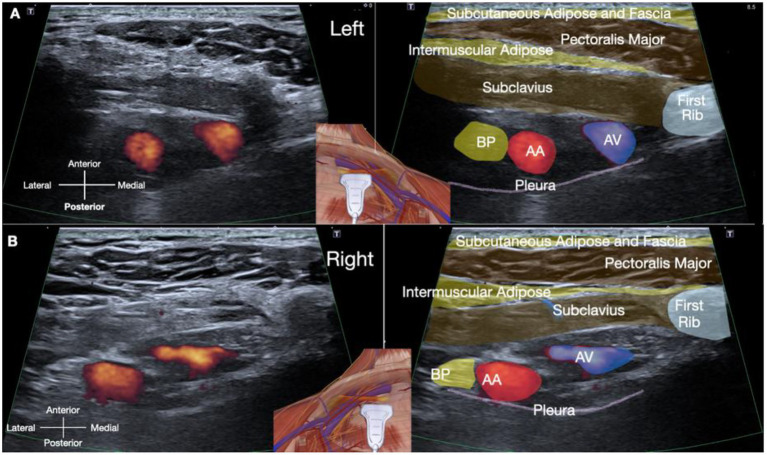
Ultrasonographic evaluation of the subclavius muscle using power Doppler. **(A)** Left side of the patient: normal subclavius muscle. The pectoralis major is superficial to the subclavius. The axillary artery (AA), axillary vein (AV), and brachial plexus (BP) are located deep (posterior) to the subclavius. Medially, the subclavius attaches to the first rib. Normal muscle bulk and preserved echotexture are observed. Color shading on the image delineates the boundaries of key infraclavicular structures relative to the subclavius, all of which are clearly labeled. Power Doppler imaging allows simultaneous visualization of vascular structures (AA, AV) and soft tissue. **(B)** Right side of the patient: marked atrophy of the subclavius muscle. The same anatomical relationships are preserved: pectoralis major superficially, AA/AV/BP deeply, and medial attachment to the first rib. However, the subclavius demonstrates reduced bulk, loss of normal architecture, and increased echogenicity, consistent with chronic atrophic change. Color shading again delineates the boundaries of infraclavicular structures relative to the atrophic subclavius, with all structures clearly labeled.

Power Doppler imaging in both panels enables direct comparison between the normal left and atrophic right subclavius using the same imaging modality. Abbreviations: AA, axillary artery; AV, axillary vein; BP, brachial plexus.

No significant abnormalities were identified in the scalene or pectoralis minor muscles.

### Intervention and clinical course

Ultrasound-guided hydrodissection was performed in the right infraclavicular region with particular attention to the atrophic subclavius muscle and the costoclavicular space. A total of 30 mL of 5% dextrose in water (D5W) was injected to mechanically separate the muscle from adjacent neurovascular structures under real-time ultrasound guidance (Figure 6; Supplementary Video 1). No immediate or delayed procedure-related adverse events were observed during or after the hydrodissection sessions.

**Figure 6:**
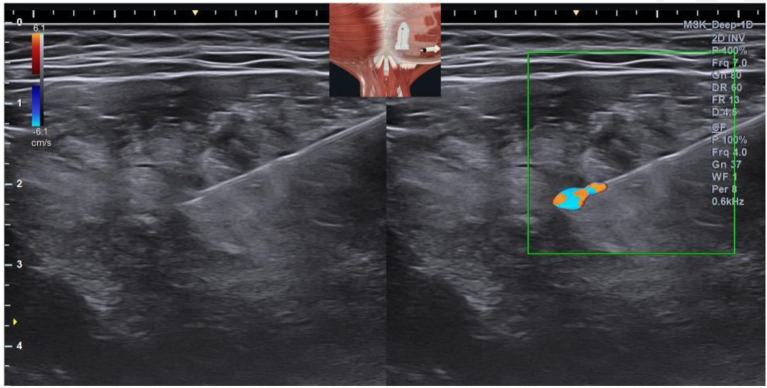
Ultrasound-guided hydrodissection of the subclavius muscle.

Live dual ultrasound images demonstrating hydrodissection of the right subclavius muscle. The patient position, transducer placement, and needle trajectory are illustrated in the inset image.

The immediate and substantial reduction in paresthesia, together with subtle bedside motor improvement following the first hydrodissection session, was not only therapeutic but also diagnostically supportive, reinforcing the subclavius muscle as a potentially clinically relevant contributor to the patient’s neurogenic symptoms.

The patient underwent six ultrasound-guided hydrodissection sessions (three weekly sessions followed by three monthly sessions). Motor strength progressively improved, with sustained symptom reduction over the follow-up period (Table 2). No concurrent treatments, medication changes, or meaningful modifications in activity level were introduced during this time. The temporal association between each session and clinical improvement supports a treatment-related effect rather than spontaneous recovery.

**Table 2:** Motor recovery following ultrasound-guided hydrodissection.

Time point	Wrist extension	Pinch grip	Elbow	Clinical status
Pre-treatment	IV	IV	IV	Persistent paresthesia
Immediately after 1st session	IV	IV	IV	>70% paresthesia reduction; subtle immediate motor improvement
3 weeks after 3rd session	IV+	IV+	IV+	Minimal residual paresthesia
1 month after 4th session	V−	V−	V−	Near-normal strength
2 months after 5th session	V	V	V	Complete symptom resolution
3 months after 6th session	V	V	V	Full functional recovery

MRC Scale: V, normal strength; IV+, mild weakness; IV, moderate weakness. Subtle immediate post-procedural motor improvement was noted clinically but was not sufficient to produce a full MRC grade change.

By the final follow-up, the patient had returned fully to work and recreational activities without recurrence of symptoms. Written informed consent for publication was obtained from the patient.

## Discussion

This case highlights an underrecognized potential anatomical contributor to nTOS: pathological involvement of the subclavius muscle. Positioned within the costoclavicular space, the subclavius muscle lies in close proximity to the brachial plexus and subclavian vessels and plays a role in stabilizing the clavicle during shoulder girdle motion. Although muscular causes of nTOS have traditionally focused on the scalene and pectoralis minor muscles, abnormalities of the subclavius muscle have received comparatively little attention in both diagnostic evaluation and interventional management.

In the present case, the patient exhibited progressive motor weakness with relatively preserved sensation, persistent proximal motor slowing on electrodiagnostic testing, and no evidence of cervical radiculopathy or distal entrapment neuropathy on imaging or nerve conduction studies. These findings collectively suggested a proximal extraforaminal neuropathic process. Targeted ultrasonographic assessment revealed marked unilateral atrophy of the subclavius muscle, a finding that is not readily appreciable on routine cross-sectional imaging and is therefore easily overlooked in standard diagnostic algorithms for nTOS.

At first glance, muscle atrophy may appear counterintuitive as a source of neurovascular compression. However, chronic pathological alteration of muscle tissue—whether due to denervation, fibrosis, or disuse—may lead to reduced compliance and abnormal tensioning of adjacent fascial planes. In the costoclavicular space, such changes may compromise the dynamic adaptability of the compartment, rendering the brachial plexus more susceptible to compression during shoulder motion or postural loading. In this context, subclavius muscle atrophy may reflect not merely loss of muscle bulk but a maladaptive structural change that could potentially contribute to dynamic neurogenic compromise, although direct evidence of causation remains speculative (12–15).

High-resolution ultrasonography was central to both diagnosis and treatment in this case. Ultrasound enabled direct comparison of bilateral subclavius muscle morphology, identification of marked asymmetry, and real-time visualization of the muscle’s relationship to adjacent neurovascular structures. Importantly, ultrasonography also facilitated precise, targeted intervention through ultrasound-guided hydrodissection. This technique allows mechanical separation of potentially adherent or noncompliant tissue planes, restoration of neural mobility, and decompression of vulnerable neural elements while avoiding the morbidity associated with surgical decompression (7, 16–19).

The immediate and progressive clinical improvement observed following hydrodissection further supports the potential clinical relevance of the subclavius muscle in this patient’s symptomatology. The temporal association between each treatment session and functional recovery, in the absence of concurrent interventions or activity modification, argues against spontaneous recovery and suggests a treatment-specific effect. While the use of 5% dextrose in water (D5W) may confer additional neuromodulatory or anti-inflammatory benefits, the primary therapeutic mechanism in this case is likely related to mechanical decompression and restoration of local tissue dynamics (8, 20, 21). From a mechanistic perspective, hydrodissection may have contributed not only by reducing local mechanical irritation but also by restoring interfascial separation and tissue gliding within the costoclavicular space (7–10).

Botulinum toxin injection has been reported as a minimally invasive option for thoracic outlet syndrome, particularly when muscular hyperactivity or spasm is believed to contribute to narrowing of the thoracic outlet. In contrast, the present case demonstrated unilateral subclavius muscle atrophy with suspected loss of compliance rather than overt muscular spasm or hypertrophy (22–24). Our therapeutic objective was therefore mechanical decompression and restoration of tissue separation and gliding within the costoclavicular space, making ultrasound-guided hydrodissection more anatomically and pathophysiologically appropriate than chemodenervation. In addition, further weakening of an already atrophic muscle was considered undesirable.

We also acknowledge that the 30 mL volume of D5W used in this case likely spread beyond the immediate confines of the subclavius muscle to affect adjacent structures within the infraclavicular region, including the pectoralis minor tendon, the costocoracoid ligament, and the surrounding fascial planes. Therefore, while we directed the hydrodissection with particular attention to the subclavius muscle and the costoclavicular space, the therapeutic effect cannot be attributed solely to decompression of the subclavius muscle. It is possible that simultaneous release of the pectoralis minor or other regional soft-tissue constraints contributed to the observed clinical improvement. Future studies using smaller injectate volumes, contrast-enhanced injectate, or comparative injection protocols may help clarify the specific contribution of the subclavius muscle versus adjacent structures.

Previous reports addressing thoracic outlet decompression have also supported the clinical relevance of the subclavius muscle within the costoclavicular space, reinforcing the concept that this structure may play a meaningful role in selected thoracic outlet syndromes. Although these reports primarily involve thoracic outlet syndrome subtypes other than neurogenic TOS, they support the broader view that the subclavius is a clinically meaningful structure rather than an incidental anatomic bystander (25, 26).

Surgical intervention remains a treatment option for refractory nTOS; however, outcomes are variable and the procedures carry inherent risks (2, 3). In contrast, ultrasound-guided hydrodissection represents a minimally invasive, repeatable, and anatomically targeted approach that may be particularly valuable in patients with focal muscular abnormalities identified on ultrasonography. This case expands the spectrum of potential muscular contributors to nTOS and suggests that the subclavius muscle warrants closer consideration during both diagnostic assessment and treatment planning.

Several limitations should be acknowledged. As a single case report, causal inference is limited, and the findings may not be generalizable to all patients with nTOS. The association between subclavius atrophy and clinical symptoms is supported by the response to hydrodissection but should be interpreted as hypothesis-generating rather than confirmatory. In addition, post-treatment electrodiagnostic confirmation was not obtained. Although the patient demonstrated substantial clinical improvement and returned to work approximately 2 months after treatment initiation, repeat nerve conduction study/electromyography was deferred because of financial burden after multiple prior diagnostic evaluations. The patient indicated a preference to reconsider follow-up testing after returning to work and regaining financial stability. The precise histopathological nature of the subclavius muscle alteration remains speculative. Future prospective studies should incorporate standardized post-treatment electrodiagnostic assessment to better correlate clinical recovery with objective neurophysiological change.

In conclusion, this case underscores the importance of comprehensive, ultrasound-based evaluation of the costoclavicular space in patients with unexplained proximal neuropathy. Ultrasound-guided hydrodissection of the infraclavicular region with attention to the subclavius muscle may represent a feasible and effective therapeutic option in selected patients with neurogenic thoracic outlet syndrome, particularly when conventional imaging and treatment approaches fail to identify or address the underlying pathology.

## Conclusion

In a patient with unexplained proximal neuropathy, ultrasonography identified focal subclavius muscle atrophy as a potential compressive contributor within the costoclavicular space. Ultrasound-guided hydrodissection of the infraclavicular region, with particular attention to the subclavius muscle and costoclavicular space, resulted in rapid and sustained clinical improvement. While the injectate volume (30 mL) likely affected adjacent structures such as the pectoralis minor and surrounding fascial planes, this minimally invasive approach may be a feasible option for selected patients with refractory nTOS. Prospective studies with larger cohorts are warranted to further define its role in the management of neurogenic TOS and to clarify the specific contribution of the subclavius muscle versus adjacent soft-tissue structures.

## Data Availability

The original contributions presented in the study are included in the article/Supplementary material, further inquiries can be directed to the corresponding authors.

## Supplementary material

SUPPLEMENTARY VIDEO 1 Live dual ultrasound images demonstrating ultrasound-guided hydrodissection of the infraclavicular region with attention to the subclavius muscle and costoclavicular space. The video shows real-time needle advancement and injectate spread within the costoclavicular space, with fluid extending into the surrounding infraclavicular fascia. Patient positioning, transducer placement, and needle trajectory are illustrated in the inset.
